# Fracture Pattern Influences Radial Head Replacement Size Determination Among Experienced Elbow Surgeons

**DOI:** 10.1007/s43465-020-00039-0

**Published:** 2020-03-18

**Authors:** Davide Cucchi, Francesco Luceri, Andrea Celli, Alessandra Menon, Raul Barco, Kilian Wegmann, Pietro Randelli, Denise Eygendaal, Paolo Arrigoni, ESSKA Elbow and Wrist Committee 2016–2018, Denise Eygendaal, Paolo Arrigoni, Luigi Pederzini, Enrico Guerra, Hakan Turan Çift, Nicolas Holzer, Boris Hollinger, Claudio Rosso, Michel van den Bekerom, Kilian Wegmann, Raul Barco, Andreas Lenich, Oskar Zupanc

**Affiliations:** 1 Department of Orthopaedics and Trauma Surgery, Universitätsklinikum Bonn, Sigmund-Freud-Str. 25, 53127 Bonn, Germany; 2 Laboratory of Applied Biomechanics, Department of Biomedical Sciences for Health, Università degli Studi di Milano, Via Mangiagalli 31, 20133 Milan, Italy; 3 IRCCS Istituto Ortopedico Galeazzi, Via Riccardo Galeazzi 4, 20161 Milan, Italy; 4 Università degli Studi di Milano, Via Mangiagalli 31, 20133 Milan, Italy; 5 Department of Orthopaedic surgery, Shoulder and Elbow Unit, Hesperia Hospital, Via Arquà, 80/A, 41125 Modena, Italy; 6 U.O.C. 1° Clinica Ortopedica, ASST Centro Specialistico Ortopedico Traumatologico Gaetano Pini-CTO, Piazza Cardinal Ferrari 1, 20122 Milan, Italy; 7 Shoulder and Elbow Unit, Hospital Universitario La Paz, Paseo de la Castellana 261, Madrid, 28046 Spain; 8 Center for Orthopedic and Trauma Surgery, University Medical Center, Cologne, Kerpenerstrasse 62, 50937 Cologne, Germany; 9 REsearch Center for Adult and Pediatric Rheumatic Diseases (RECAP-RD), Department of Biomedical Sciences for Health, Università degli Studi di Milano, Via Mangiagalli 31, 20133 Milan, Italy; 10 Department of Orthopaedic Surgery, Academic Medical Center, University of Amsterdam, Amsterdam, The Netherlands; 11 Upper Limb Unit, Department of Orthopaedic Surgery, Amphia Hospital, Breda, The Netherlands

**Keywords:** Elbow, Radial head, Fracture, Replacement, Prosthesis, Agreement, Anatomical study

## Abstract

**Background:**

Correct sizing is challenging in radial head replacement and no consensus exists on the implant’s optimal height and width to avoid elbow stiffness and instability. Studies exists, suggesting how to appropriately choose the implant size, but the manner by which the fracture pattern influences the surgeons’ operative choices was not investigated.

**Methods:**

The radial heads of four fresh-frozen cadaveric specimens were excised, measured, and fractured to simulate four patterns: three fragments (A); four fragments (B); comminuted (C); comminuted with bone loss (D). Nine examiners were asked to indicate first the maximum diameter of the radial heads with the help of dedicated sizing dishes and then the appropriate implant size with trial implants. Accuracy and precision were determined. A coefficient of variation was calculated and agreement was evaluated with the Bland–Altman method.

**Results:**

Accuracy and precision of radial head diameter estimation with dedicated sizing dish were 96.73% and 93.64%, (best pattern, D; worst, C). Accuracy and precision of radial head diameter estimation with trial implants were 99.71% and 90.66% (best pattern, A; worst, D). Frequent modifications occurred between the initial radial head size proposal based on the sizing dish and the radial head size chosen after use of the trial implants (47.2%).

**Conclusions:**

Diameter estimation of radial heads with dedicated sizing dishes may be underestimated in comminuted fractures; when bone loss is present, this may lead to an overestimation, especially when using trial implants. Care is essential to determine the optimal size of the implant and to avoid overlenghtening and oversizing, which can be responsible for implant failure.

**Level of Evidence:**

Basic Science Study.

**Clinical Relevance:**

Knowledge of the manner by which the fracture pattern influences radial head replacement size estimation can help preventing overlenghtening and oversizing during this procedure.

## Introduction

Radial head (Rh) fractures are among the most common elbow fractures, occurring in up to 20% of all elbow injuries [1]. Rh prosthetic replacement (RhR) is indicated for comminuted Rh fractures in association with other elbow fractures, with lesions of the interosseous membrane or with elbow instability [2–5]. This surgical treatment can provide satisfactory results at long-term follow-up [6–9].

The goal of an RhR is to substitute the fractured Rh with an implant able to withstand the loads and transmit the forces from the forearm and to provide lateral column stability against varus and valgus forces, without interfering with elbow range of motion [10].

A paradigm to obtain a successful RhR is that the implant must be positioned at the same height of the native Rh, and it must replicate its thickness and diameter [11].

An excessively small or short implant may cause residual elbow instability. On the contrary, overlengthening can increase the pressure on the humeral condyle and induce cartilage degeneration, pain and stiffness, whereas oversizing can increase the interosseous membrane tension [11–20]. The most common cause of RhR failure is malposition of the implant, in particular in overlengthening [11].

Although several biomechanical and clinical studies exists, suggesting how to appropriately choose the implant size for a RhR, to our knowledge only one study has evaluated the effect of fracture comminution on the radial head measurement accuracy [21] and no studies have investigated how different surgeons would approach the same fracture and how the fracture pattern may influence their operative choices.

To fill this gap in the currently available literature, this study was designed with the aim to evaluate how the fracture pattern influences the reproducibility of on-table Rh diameter determination and on-site RhR sizing among a panel of experienced elbow surgeons.

## Materials and Methods

**Fig. 1:**
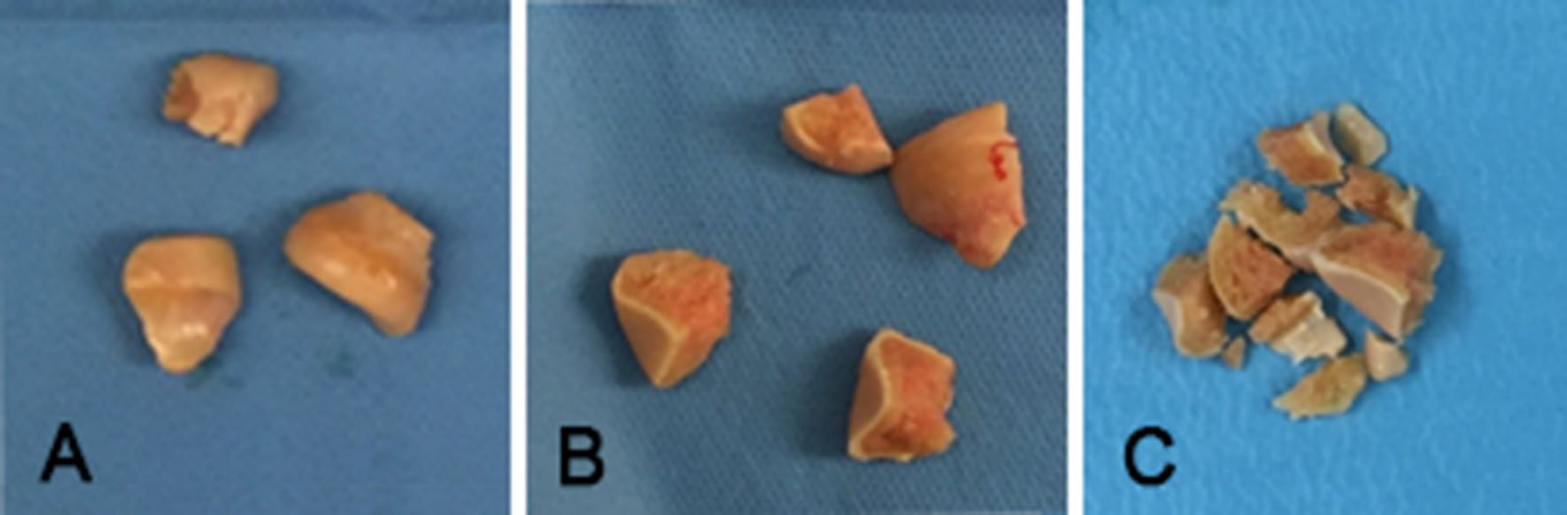
Different fracture patterns considered for this study: three-fragment fracture (**a**); four-fragment fracture (**b**); comminuted fracture (**c**). Pattern D is has the same appearance of pattern C, but some fragments were removed to simulate bone loss

Four fresh-frozen non-paired cadaveric upper limb specimens were dissected using a standard Kocher approach. Before investigation, care was taken to evaluate the specimens for visible signs of previous trauma, gross instability, arthritis or deformity. An osteotomy of the radial neck was performed 10 mm from the top of the Rh, perpendicular to the radial shaft. Subsequently, the Rh maximum diameters were first estimated with dedicated sizing dish with circular cutouts and then measured with a graduated calliper; similarly, the minimum diameters were measured with a graduated calliper. Two Rhs were then fractured to simulate a three-fragment fracture (A) and a four-fragment fracture (B) as previously described by Abdulla et al. [21]; the third Rh was fractured to simulate an extremely comminuted pattern (C); the last one was prepared to simulate a comminuted fracture with bone loss (D) (Fig. 1).

Subsequently, nine independent examiners with experience in RhR surgery (more than 10 procedures/year) were asked to complete the following tasks for each of the four Rhs:

*Task (1) On-table Rh diameter determination*: Indicate the maximum diameter of the excised Rh with the help of a dedicated sizing dish with circular cutouts (Acumed^®^ Anatomic RhR), but without possibility of viewing the corresponding specimen. This parameter was measured as the smallest circular cutout in which the reconstructed Rh fitted smoothly, herewith indicating the maximum diameter of the Rh.

**Fig. 2:**
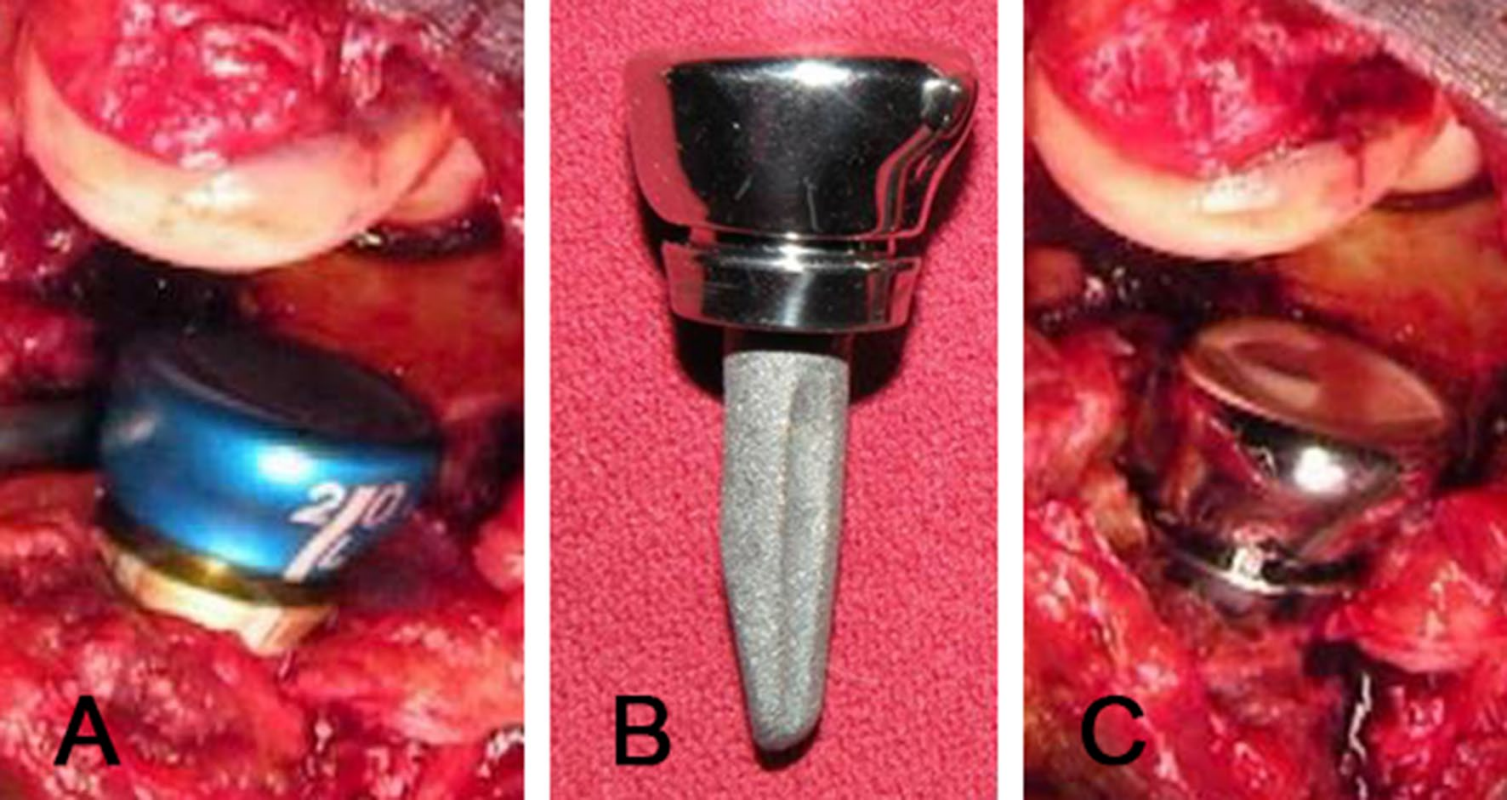
Clinical pictures of the radial replacement system used: intra-operative picture with definitive stem with trial head (**a**); assembled prosthesis (**b**); intra-operative picture with definitive components (**c**)

*Task (2) On-site RhR sizing*: Indicate the appropriate size (diameter and height) for a RhR by the use of trial implants, with possibility to test them on the corresponding specimen. An anatomic, nonaxisymmetric, modular RhR system was used (Acumed^®^ Anatomic RhR—Fig. 2). This design was chosen, since it reproduces the ellipsoid and conical shape of the native Rh, which has been demonstrated to provide improved contact mechanics [22–24]. The chosen diameter was then compared to the diameter of the native Rh

To prevent radial neck damage due to repeated trials by multiple examiners, the diameter of the prosthetic stem was chosen from the first examiner and the prosthetic stem was then implanted and left in place for the whole duration of the study: each following examiner was allowed to change the trial collar component to adjust the implant height and the head diameter to adjust its size. Each surgeon was left free to chose the anatomical landmarks or reference points and to perform the intra-operative fitting and stability tests he/she was most confident with to evaluate the appropriate component size.

Institutional approval of the study protocol was obtained by the Nicola’s Foundation & ICLO Research Center (ID10605).

### Statistical Analysis

Statistical analysis (A.M.) was performed using GraphPad Prism v 6.0 software (GraphPad Software Inc.). The Shapiro–Wilk normality test was used to evaluate the normal distribution of the sample. Continuous variables were expressed as median and interquartile (IQ) range (first and third quartiles) or as mean ± standard deviation (SD), as appropriate. Means, standard deviations, and 95% confidence intervals [CI95%] were calculated. Accuracy (defined as the closeness of the estimated measurements to the diameter of the Rh measured prior to fracture)and precision (defined as the closeness of the set of estimates among themselves) were determined as functions of mean and SD for each sizing method and each distinct fracture pattern, and then as cumulative value for all esteems with the same method.

The differences among the four different patterns for continuous variables were proved with an unpaired Student’s *t* test or Mann–Whitney test according to the characteristics of the data distribution. Friedman non-parametric test was used to assess within-group difference and to compare measurements repeated in different conditions.

A coefficient of variation (CoV), defined as the ratio of the standard deviation to the mean, was calculated to determine agreement among observers and intra-observer variability. Agreement between results for pairs of sizes obtained from tasks 1 and 2 and effective sizes was evaluated by use of the Bland–Altman method [25]. The Limits of Agreement (LOA) were defined as the 95% confidence interval (CI) of the mean difference between the sizes. For all analyses, the significance level was set at *p* value lower than 0.05.

## Results

Complete sets of measurements were obtained for both tasks, from all nine examiners.

### Task (1) On-Table Rh Diameter Determination

**Fig. 3:**
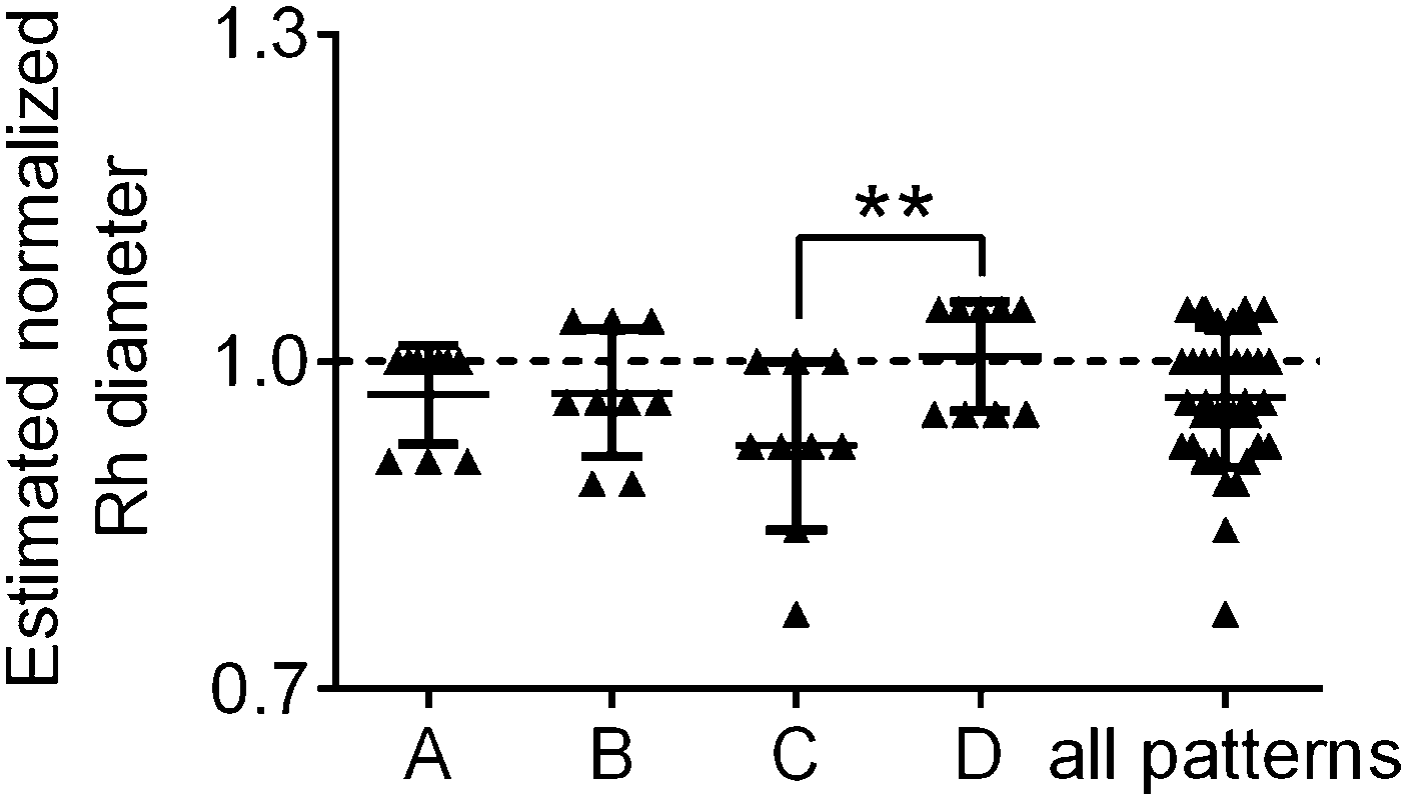
Graphic representation of the results of Task (1) On-table radial head diameter determination: box-plot showing the radial head diameter as estimated with the dedicated sizing dish, normalized for the maximum diameter of the native radial head. The dashed line indicates the target of the task (maximum diameter of the radial head)

The overall accuracy and precision of Rh diameter estimation with the dedicated sizing dish were 96.73% and 93.64%. Accuracy was best for pattern D and worst for pattern C. Precision was best for pattern A and worst for pattern C (Table 1). When comparing accuracy for different patterns, a significant difference was registered only between pattern C and pattern D (*p* = 0.0054) (Fig. 3). Overall variability among observers was 6.16%; in subgroup analysis, variability was greater in pattern C (Table 1).

**Table 1:** Summary of the study results

	Overall (%)	A (%)	B (%)	C (%)	D (%)
Radial head maximum diameter		22 mm	27 mm	26 mm	21 mm
Task 1					
Accuracy	96.73	96.97	97.12	92.31	99.47
Precision	93.64	95.46	94.21	92.31	94.98
CoV	6.16	4.69	5.96	8.33	4.99
Task 2					
Diameter					
Accuracy	99.71	96.64	94.02	95.24	93.77
Precision	90.66	90.20	92.52	92.93	92.37
CoV	9.37	9.49	7.95	7.42	7.18
Height					
CoV	57.80	43.30	79.51	54.08	36.08

### Task (2) On-Site RhR Sizing

**Fig. 4:**
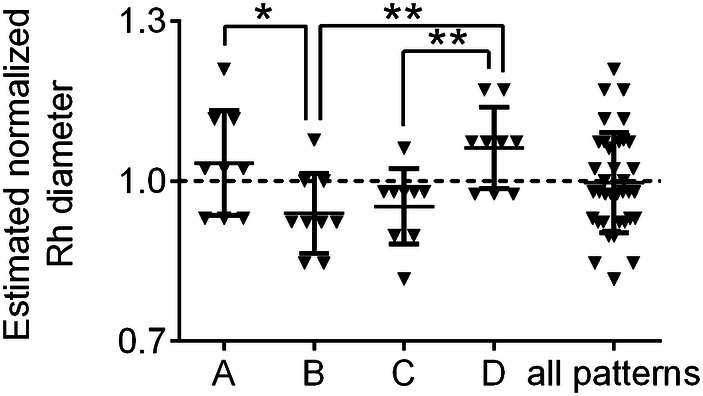
Graphic representation of the results of Task (2) On-site radial head replacement sizing: box-Plot showing the radial head diameter as estimated with the trial implants, normalized for the average diameter of the native radial head. The dashed line indicates the target of the task (average diameter of the radial head)

The overall accuracy and precision of Rh diameter estimation with the trial implants were 99.71% and 90.66%. Accuracy was best for pattern A and worst for pattern D. Precision was best for pattern C and worst for pattern A (Table 1). When comparing accuracy for different patterns, a significant difference was registered between pattern A and B (*p* = 0.0372), B and D (*p* = 0.0034), C and D (*p* = 0.0059) (Fig. 4). Overall variability among observers was 10.3%; in subgroup analysis, variability was greater in pattern A (Table 1).

For Rh height, overall variability among observers was 57.80%; in subgroup analysis, variability was greater in pattern B (Table 1).

### Modifications Between On-table Rh Diameter Determination and On-site RhR Sizing

Frequent modifications occurred between the initial Rh size proposal based on the dedicated sizing dish and the Rh size chosen after use of the trial implants on the specimens (47.2%). These modification occurred more frequently in pattern B (66.7%) (Table 2).

**Table 2:** Frequency of the modifications occurred between the initial Rh size proposal based on the dedicated sizing dish and the Rh size chosen after use of the trial implants

Overall (%)	A (%)	B (%)	C (%)	D (%)
47.22	33.33	66.67	55.56	33.33

**Fig. 5:**
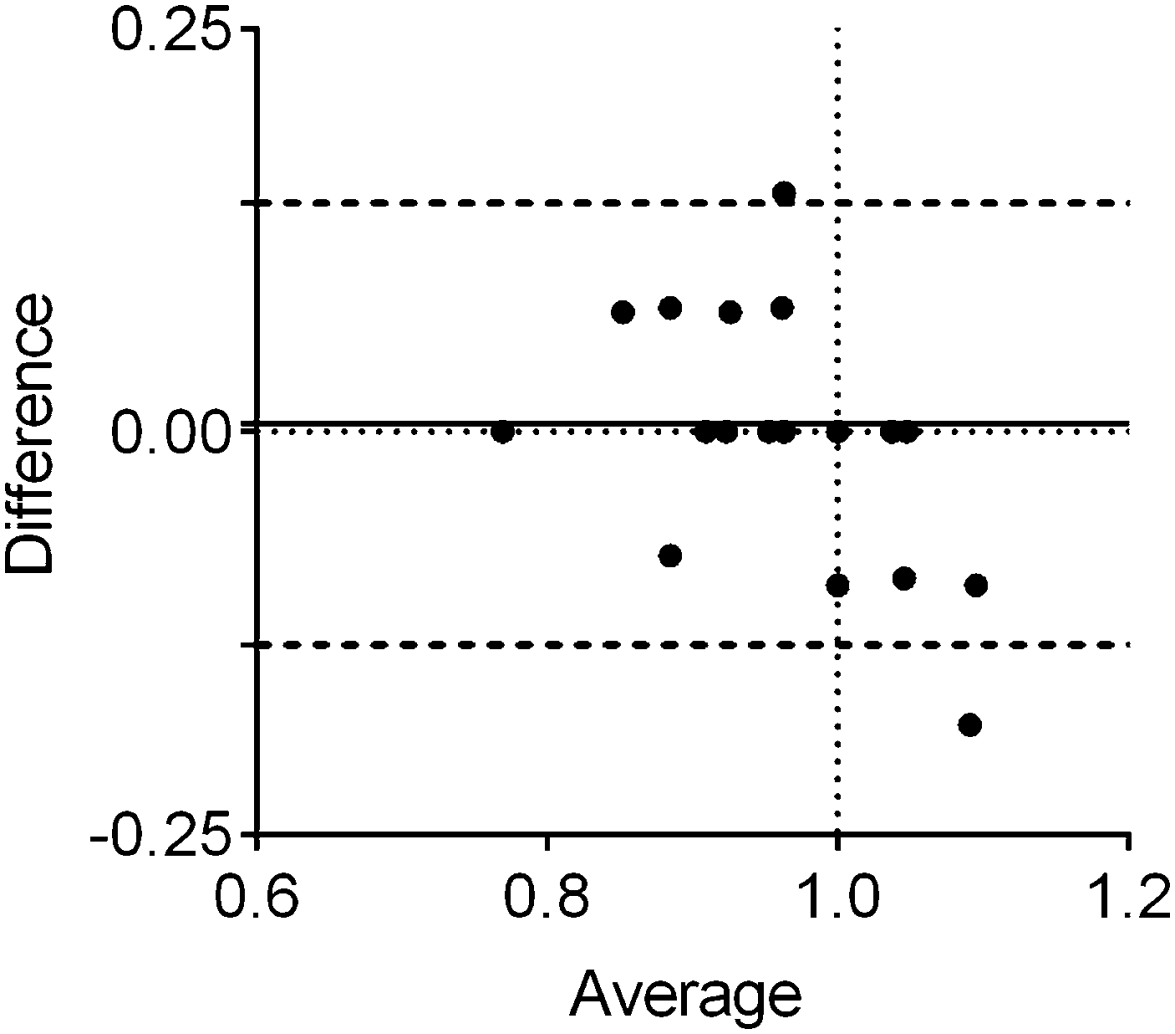
Bland–Altman plot depicting agreement of values between the radial head diameter estimated with the sizing dish (Task 1) and the radial head size estimated with the trial implants (Task 2). *Y* axis: difference between normalized Rh diameters estimated with the sizing dish and with trial implants. *X* axis: average between normalized Rh diameters estimated with the sizing dish and with trial implants. The vertical pointed line indicates the native radial head diameter. The horizontal pointed line indicates 0 difference, the solid line represents the mean difference in measurements and two dotted lines represent the 95% confidence intervals (CIs) for the mean difference (LOA)

The agreement between the initial Rh size proposal based on the dedicated sizing dish and the Rh size chosen after use of the trial implants was acceptable. Especially by Rh with smaller diameters, the surgeons tended overestimate them with the sizing dishes and then chose a smaller implant; the Rh with larger diameters showed the opposite tendency (Fig. 5).

## Discussion

The main findings of this study are that accuracy and precision in on-table Rh diameter determination are worse in Rh comminuted fractures without bone loss and that the variability between surgeons when measuring fractures with this pattern increases as compared to other fracture patterns. On the other hand, a lower accuracy in on-site RhR sizing was registered for comminuted fractures with bone loss, for which a tendency to an overestimation appeared.

Moreover, frequent modifications occurred between the Rh diameter determined using the dedicated sizing dish and the Rh size chosen after use of the trial implants on the specimens.

Our results demonstrated also that the smaller diameters tended to be slightly overestimated by the sizing dishes and underestimated by the trial implants, whereas the larger diameters showed the opposite tendency. Great care appears to essential to determine the optimal size of the implant and to avoid overlenghtening and oversizing, which can be responsible for implant failure.

Several options have been described to treat a comminuted Rh fracture, including conservative treatment, an “on-table” reconstruction [26], excision [27], interposition with anconeus [28, 29], bone grafting [30] and RhR [31, 32].

Over the years, RhR evolved to closely replicate the physiological radiocapitellar tracking, reproducing the mechanical functions of the native Rh: shear the forces passing through the elbow and stabilize it while allowing flexion and pro-supination movements [5]. This stabilizing role of the RhR is particularly important the presence of an injury to the collateral ligaments or a coronoid fracture: in these cases, the Rh, which is considered a secondary stabilizer in the uninjured elbow [33], gains a fundamental importance [4, 34, 35].

Two fundamental principles must be applied to select a suitable Rh implant: the implant must be positioned at the same height of the native Rh, and it must replicate its diameter [11].

The negative drawbacks of an inappropriate implant height have been extensively described: a “low” implant may cause residual elbow instability, whereas “high” implant, (overlengthening) decreases the tension on the interosseous membrane and can excessively increase the pressure on the humeral condyle, rapidly inducing cartilage degeneration on both the lateral and medial side, and subsequent pain, limitation in elbow range of motion and post-traumatic arthritic changes [12–18].

Only few studies have investigated the role of the implant diameter, indicating that this parameter could alter point-loading at the rim of the implant on the capitellum and may influence the interosseous membrane tension and tension of the lateral collateral ligament complex [11, 19, 20].

Since the correct choice of diameter and height of a RhR plays a relevant role in this surgery, numerous techniques, based on radiological or anatomical landmarks, have been described to assist the surgeon in selecting the most appropriate of Rh size to prevent overlengthening and oversizing [13, 17].

A preoperative radiograph of the contralateral elbow can provide insight into the native Rh anatomy to estimate the implant sizes prior to surgery. When considering this strategy, Vaquero-Picado et al. recently suggested that, although the measure of the Rh diameter is the most consistent among different observers (ICC: 0.904), the humeral condyle diameter can better predict the final component diameter, still maintaining an acceptable interobserver concordance (ICC: 0.888). This study revealed also that the estimation of the Rh implant height on lateral radiographs is poorly reproducible among surgeons (ICC: 0.443) [36]. CT scans, when available, can provide additional information on Rh anatomy and guide the surgeon towards the appropriate size planning [13, 36–38].

During surgery, other adjunctive strategies are available to verify the correct implant size. Reconstruction on the operating table of the native head (as a jig saw puzzle) can help confirming that all the intra-articular fragments have been removed and can provide a guidance about the best implant diameter: once the Rh has been reconstructed, its diameter can be determined with graduated callipers or dedicated sizing dishes. In this situation, the measurement of the minimum and maximum diameters showed excellent intra-observer and inter-observer reliability, whereas the measurement of the inner articular dish diameter has been associated to lower reproducibility, perhaps due to the variable shape of the inner articular dish or to difficulty in determining, where the dish actually starts [20]. When measuring diameters with sizing trays, it should be noted that smallest circular cutout in which the reconstructed head fits indicates the maximal diameter of the radial head. Since oversizing can be responsible for implant failure, if there is doubt between two sizes, the smaller size should be selected [39]. Abdulla et al. investigated the effect of fracture comminution on radial head measurement accuracy, showing that the measurements of the maximum and minimum diameter were more reliable than the measurement of the articular dish for diameter sizing of intact and comminuted radial heads. In this study, the reliability did not change significantly between measurements performed on two-fragment, three-fragment, and four-fragment fractures [21]. The present study extends these findings to extremely comminuted fracture patterns and additionally evaluates the effect of adjusting the Rh implant size based on the fit of the implant to the elbow of a complete specimen.

The results confirm that the fracture pattern has an influence not only on the surgeons’ estimation but also on the implant choice. Surprisingly, the worst figures for accuracy and precision were recorded for the comminuted fracture pattern without bone loss (C) and not, as expected, in the comminuted fracture pattern with bone loss (D). A significantly smaller diameter was, in facts, estimated for pattern C, as compared to pattern D. These results do not have a clear explanation: we suppose that comminution leads in both patterns to a slight underestimation of the maximum Rh diameter; however, in our study setting, the knowledge that a bone loss was present might have influenced the surgeons’ estimation, leading to a significantly higher value. As a consequence, when estimating the Rh with dedicated sizing dishes in comminuted fractures, the surgeon should consider that this pattern may lead to underestimate the Rh diameter.

Nevertheless, since oversizing can be responsible for implant failure, if there is doubt between two sizes during reconstitution on the operating table of the native head, the smaller size should be selected; in this case, adequate tensioning of the annular ligament is essential to avoid ballottement of the prosthetic head [39].

The relationship between the trial implant and intra-operative anatomical landmarks may also help in identifying the correct Rh size. Doornberg et al. [13] found on CT reconstruction that the lateral edge of the coronoid articular surface is a reproducible landmark, and suggested to place the articular surface of the implant slightly more proximal than this landmark, whereas Müller et al. [40] used trial stems to determine the size of the implant and concluded recommending to obtain 0.5 mm between the head of the replacement and the capitulum humeri. The lesser sigmoid notch of the ulna, which has been demonstrated to be a reproducible landmark to choose the implant height [41], has also been proposed as a possible landmark to assist in choosing the correct Rh diameter, but has been associated to poor interobserver reliability both intra-operatively and on preoperative CT scans [20].

In our study, each investigator was left free to use the reference system he/she felt most confident with. Interestingly, the results revealed that among these experienced surgeons a great variability in Rh size choice exists, especially for Rh height. When considering the implant size, a lower accuracy in on-site Rhr sizing was registered for comminuted fractures with bone loss. Moreover, a tendency to an overestimation appeared for this fracture pattern, which could be related to the knowledge that a bone loss was present in the specific study case. Our results demonstrate that the smaller diameters tended to be slightly overestimated by the sizing dishes and underestimated by the trial implants, whereas the larger diameters showed the opposite tendency. 47.2% modifications occurred between the Rh size proposal based on the dedicated sizing dish and the Rh size chosen after use of the trial implants on the specimens. These modifications occurred more frequently in four-fragment fracture (66.7%).

Intra-operative fluoroscopy is another helpful tool to evaluate the implant sizes: this may be used to evaluate the symmetric appearance of the medial and lateral sides of the humero-ulnar joint space and to verify the alignment of the implant on the ulnar notch. Kim et al. [42] evaluated 9 cadaveric specimens found that a perfectly anatomic RhR with an articular surface that is completely aligned with the articular surface of the coronoid process appears slightly overlengthened by approximately 2 mm in anteroposterior radiographs. Fluoroscopic control of the ulnar variance in the ipsilateral wrist has been used to help in detecting oversizing of monopolar Rh prostheses [43], but these results were not confirmed with multipolar prostheses [14], perhaps due to compensatory movements within the radiocapitellar joint allowed by multipolar designs [44].

Limitations of this study include that it is an anatomical study on a limited number of cadaveric specimens: this is not suitable to reproduce the soft tissue characteristics of the living subject, cannot directly predict clinical outcomes and could amplify bias related to anatomical variants. However, to minimize possible bias related to the approach or to repeated tissue trauma from different investigators, all approaches were conducted by the same surgeon and the prosthetic stem was left in place for the whole duration of the study.

Moreover, a single prosthetic model was used (anatomic, modular, nonaxisymmetric Rh hemireplacement); other systems may perform differently and these results may then not be representative for all different RhR models available.

Finally, this experimental setting was designed to specifically evaluate the interobserver reproducibility of diameter and height determination: the consequences of these choices on the radiocapitellar contact area and pressure, on the radial length and on the tension of the interosseous membrane were not investigated.

## Conclusions

Sizing of RhR is challenging after comminute Rh fractures; there is no consensus on the optimal height and width of the implant to avoid stiffness or instability of elbow. When estimating the Rh with dedicated sizing dishes in comminuted fractures, the surgeon should consider that this pattern may lead to underestimate the Rh diameter. On the other hand, when bone loss is present, this may lead to an overestimation of the Rh implant size, especially when using trial implants. Great care is essential to determine the optimal height of the implant and to avoid overlenghtening and oversizing, which can be responsible for implant failure. For this reason, if there is doubt between two sizes during reconstitution on the operating table of the native head, the smaller size should be selected.
